# Antibody-Dependent Enhancement: A Challenge for Developing a Safe Dengue Vaccine

**DOI:** 10.3389/fcimb.2020.572681

**Published:** 2020-10-22

**Authors:** Rahul Shukla, Viswanathan Ramasamy, Rajgokul K. Shanmugam, Richa Ahuja, Navin Khanna

**Affiliations:** Translational Health Group, Molecular Medicine Division, International Centre for Genetic Engineering and Biotechnology, New Delhi, India

**Keywords:** dengue, dengue virus (DENV), dengue vaccine, live attenuated vaccine, Dengvaxia, antibody-dependent enhancement (ADE), virus-like particle

## Abstract

In 2019, the United States Food and Drug Administration accorded restricted approval to Sanofi Pasteur's Dengvaxia, a live attenuated vaccine (LAV) for dengue fever, a mosquito-borne viral disease, caused by four antigenically distinct dengue virus serotypes (DENV 1-4). The reason for this limited approval is the concern that this vaccine sensitized some of the dengue-naïve recipients to severe dengue fever. Recent knowledge about the nature of the immune response elicited by DENV viruses suggests that all LAVs have inherent capacity to predominantly elicit antibodies (Abs) against the pre-membrane (prM) and fusion loop epitope (FLE) of DENV. These antibodies are generally cross-reactive among DENV serotypes carrying a higher risk of promoting Antibody-Dependent Enhancement (ADE). ADE is a phenomenon in which suboptimal neutralizing or non-neutralizing cross-reactive antibodies bind to virus and facilitate Fcγ receptor mediated enhanced entry into host cells, followed by its replication, and thus increasing the cellular viral load. On the other hand, antibody responses directed against the host-cell receptor binding domain of DENV envelope domain-III (EDIII), exhibit a higher degree of type-specificity with lower potential of ADE. The challenges associated with whole DENV-based vaccine strategies necessitate re-focusing our attention toward the designed dengue vaccine candidates, capable of inducing predominantly type-specific immune responses. If the designed vaccines elicited predominantly EDIII-directed serotype specific antibodies in the absence of prM and FLE antibodies, this could avoid the ADE phenomenon largely associated with the prM and FLE antibodies. The generation of type-specific antibodies to each of the four DENV serotypes by the designed vaccines could avoid the immune evasion mechanisms of DENVs. For the enhanced vaccine safety, all dengue vaccine candidates should be assessed for the extent of type-specific (minimal ADE) vs. cross-reactive (ADE promoting) neutralizing antibodies. The type-specific EDIII antibodies may be more directly related to protection from disease in the absence of ADE promoted by the cross-reactive antibodies.

## Introduction

Daily more than a million people are infected with any of the four distinct serotypes of dengue viruses (DENV-1, −2, −3, and, −4). The world needs a dengue vaccine for all age groups, regardless of whether they may, or may not have been previously exposed to one of the four dengue viruses. In 2019, Sanofi Pasteur's Dengvaxia, a live attenuated tetravalent dengue vaccine, which does not give complete protection, after a three-dose regimen spread over 1 year (Arredondo-García et al., 2018; Thomas and Yoon, 2019), was granted a limited approval by the United States Food and Drug Administration. This approval was for its use in 9–16 year-old children with laboratory-confirmed previous dengue infection, living in dengue-endemic areas (United States Food & Drug, 2019). Efficacy trials of Dengvaxia in several dengue-endemic countries of Asia and Latin America, in ~35,000 2–16 year-old children (Sabchareon et al., 2012; Capeding et al., 2014; Villar et al., 2015), showed that vaccine efficacy (VE) i.e., the capacity to prevent symptomatic virologically confirmed dengue (VCD), varied by serotype, and was the lowest against DENV-2. Overall VE was 65.6% (95% CI 60.7–69.9) in 9–16 year olds, while in 2–8 year olds it was 44.6% (95% CI 31.6–55), at 2 years following administration of the first dose of the vaccine. At 3 years post-dose 1, vaccinated children in the 2–5 year age group, were found to be nearly 8 times likely to be hospitalized for severe dengue, compared to children in the placebo group (Hadinegoro et al., 2015). VE was subsequently found to be related to the pre-vaccination serostatus of the trial subjects. While VE against VCD at 2 years post-dose 1 was 76% (95% CI 64–84) in >9 year-old children, who had been exposed to dengue infection before vaccination (seropositive), it was only 39% (95% CI−1 to 63) in children who were dengue-naïve (seronegative) at the beginning of the trial (Sridhar et al., 2018). Long-term follow-up studies until 5 years reveal that in seronegative recipients, there is increased risk of severe dengue from the 3rd year onwards, post-dose 1. Clearly, developing a safe and efficacious dengue vaccine constitutes quite a formidable challenge. Several unique factors, associated with the biology, and pathogenesis of dengue, taken together with lessons of the Dengvaxia experience, necessitates exploring alternate dengue vaccine development options. There are additional whole virus-based dengue vaccines in advance stages of clinical trials (Clinicaltrials.gov, 2020; Tricou et al., 2020). Moreover, a few recombinant dengue vaccine candidates are also at various stages of development (Vannice et al., 2015; Swaminathan and Khanna, 2019; Deng et al., 2020). It is great that the pipeline of dengue vaccines continue to increase. A safe and effective dengue vaccine could soon become a reality.

## Dengue: The Virus and the Disease

DENVs contain a positive sense RNA genome within a glycoprotein shell and are members of the Flaviviridae family, which includes other human pathogenic viruses such as yellow fever virus (YFV), Japanese encephalitis virus (JEV), and Zika virus (ZIKV) (Pierson and Diamond, 2013; Poland et al., 2018). The DENV genome, which is similar in organization to that of the other flaviviruses, encodes ten viral proteins, three of which are structural: the capsid (C), envelope (E), and membrane (M) and the remaining non-structural (NS): NS1, NS2A, NS2B, NS3, NS4A, NS4B, and NS5. Two of the structural proteins, the envelope (E) and the pre-membrane (prM) proteins, form the glycoprotein shell of the virus. The E protein is organized into three discrete domains, envelope domain I (EDI), EDII, and EDIII (Modis et al., 2003). The EDI participates in the confirmation changes required for virus entry. The EDII has a fusion loop (FL) which is required for fusion with the host membrane, as a prelude to release of the DENV RNA into the cytosol of the infected cell, and EDIII is believed to be responsible for interaction with the host-receptor molecule (Crill and Roehrig, 2001; Hung et al., 2004; Huerta et al., 2008; Hidari and Suzuki, 2011; Modis, 2014). The prM protein helps mask the FL of EDII to avoid premature fusion and release into cytosol during virus maturation within the infected cell. The immature virus is decorated with spikes of trimers of prM-E dimers. As a final step in virus maturation in the trans-Golgi network, prM is cleaved by host-encoded furin, leaving a peptide (pr) still covering the FL. Upon secretion to the outside of the infected cell, pr peptide dissociates from the virion, which is now fully mature and smooth (Screaton et al., 2015).

**Figure 1:**
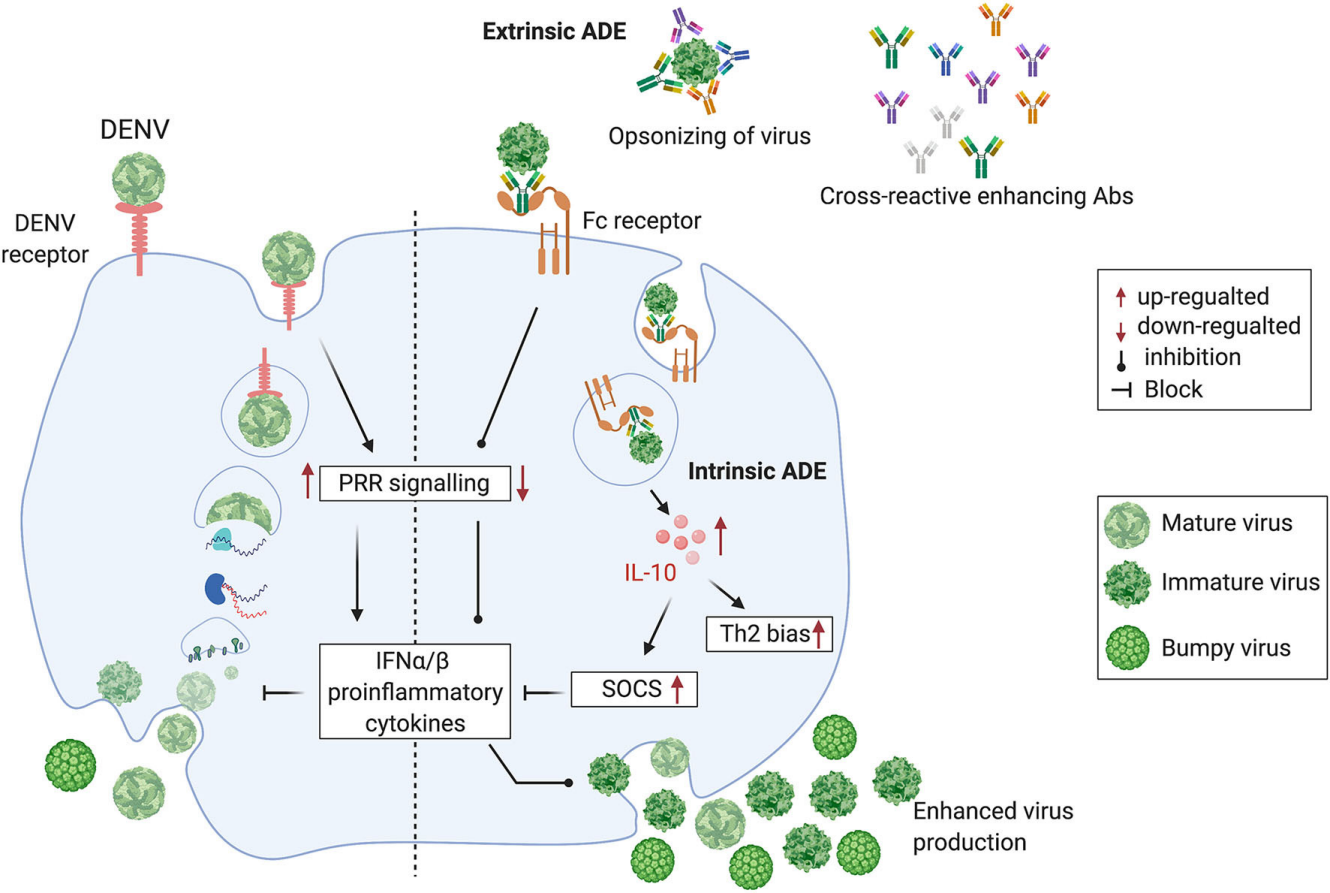
Cross-reactive, prM and fusion-loop Abs facilitate immature DENV entry through Fcγ receptor and mediate enhanced DENV replication by following the intrinsic ADE pathway. **(Left half)** DENV attaches to a host cell surface and is endocytosed followed by virus-endosomal membrane fusion leading to the release of viral genome. Post-release, viral RNA is translated and the viral genome is replicated. Virus assembly occurs on the surface of the endoplasmic reticulum, and immature viral particles mature into their infectious form in the Golgi network. These mature viruses are then released from the cell and ready to infect other cells. **(Right half)** Cross-reactive Abs bind with immature non-infectious particles turning into infectious virus-Ab immune complexes (V-Ab IC) which then bind with the Fc receptor bearing cells. This assembly down-regulates the DENV-specific pattern recognition receptor (PRRs) signaling, inhibits type I interferon (IFNα/β) release and activates production of Interleukin-10 (IL-10) which causes up-regulation of Suppressor of Cytokine Signaling (SOCS) family. Henceforth, controlled mature DENV production is lost, resulting into manifold increase in wide-range of immature viruses which leads to the extrinsic-ADE pathway by infecting other cells via binding with cross-reactive Abs (Figure is adapted from Halstead et al., 2010 under Copyright license, # 4852311472038 and generated in Biorender.com).

In most clinically apparent cases, DENVs cause a self-limiting febrile illness known as dengue fever. However, a small proportion of DENV infections cause severe dengue. This is a potentially fatal form of dengue disease, characterized by increased capillary permeability leading to plasma leakage and shock (Simmons et al., 2012). Severe dengue has often been associated with sequential infection with DENVs of different serotypes. It has been proposed that antibodies to a given DENV serotype induced during a first infection, bind to, but do not neutralize, a different DENV serotype, encountered during a subsequent infection (Tsai et al., 2015). In fact, the cross-reacting or neutralizing antibodies at suboptimal levels facilitate increased uptake of the non-neutralized DENV into monocytes and macrophages, considered to be the *in vivo* sites of DENV replication, via their Fcγ receptors. This phenomenon is termed antibody-dependent enhancement (ADE) (Halstead and O'Rourke, 1977; Dejnirattisai et al., 2010). The DENV-induced prM and fusion-loop Abs facilitate immature DENV entry through Fcγ receptor and mediate enhanced DENV uptake into cells, facilitating subsequent increased viral replication (Extrinsic ADE). The DENVs immune complexed with these antibodies upon entry via Fcγ receptor results in the suppression of intracellular cytokine signaling, causing a favorable environment for enhanced replication of DENVs (Intrinsic ADE). The Fcγ receptor-mediated DENV entry to host cells seems to be ten times more productive than the DENV entry through its host-cell receptor, thus increasing the DENV load (Halstead et al., 2010). On the contrary, the host cell receptor-mediated DENV entry induces PRR (Pattern recognition receptors) signaling, resulting in the suppression of DENV viral replication, thus controlling the DENV load (Figure 1).

There are several *in vitro* and *in vivo* studies that support ADE mediated enhanced disease outcome. Dengue virus-induced sera or monoclonal antibodies increase the DENV infection of Fcγ receptor-bearing cells (Goncalvez et al., 2007). Non-human primates passively immunized with dengue virus antibodies promoted higher level of viremia as compared to dengue virus infection in the absence of antibodies (Muhammad Azami et al., 2020). A similar outcome was reported earlier using AG129 mouse model, where, passively transferred DENV-induced antibodies enhanced non-lethal DENV infection into a lethal infection, associated with vascular leakage and cytokine storm (Watanabe et al., 2015).

Apart from this, two longitudinal clinical studies from Thailand and Nicaragua (Katzelnick et al., 2017; Salje et al., 2018) evaluated the risk of severe dengue disease following primary and secondary infection. These clinical studies provide strong evidence that highest risk of severe dengue is associated with low levels of pre-existing dengue antibodies. These studies have revealed that low level pre-existing antibody levels are correlated with an increased likelihood of severe dengue disease only during secondary heterotypic infections (Katzelnick et al., 2017). However, ADE is not observed during secondary homotypic infection. This is because highly neutralizing type-specific Abs elicited during primary infection can neutralize a secondary homotypic infection even at low Ab concentrations, preventing the incidence of ADE (Ripoll et al., 2019). In homotypic infection, neutralization is determined by type-specific antibodies and ADE is limited to a very low level of type-specific antibody concentration. During heterotypic infection both type-specific and cross-reactive antibodies contribute to virus neutralization, but at a lower efficiency. Molecular simulations have shown that rough form of the virus become particularly pathogenic in case of heterotypic infection, as the neutralization is determined by cross-reactive Abs (Ripoll et al., 2019).

## Genetic Diversity

There are four antigenically distinct serotypes of DENVs, differing in amino acid (aa) identity of their E proteins by ~40%. Each of these four DENV serotypes can cause dengue disease ranging from mild to severe manifestations (Simmons et al., 2012). Within each serotype there are several genotypes, with genomic sequences differing by as much as 6% (Rico-Hesse, 2003). Further, when DENVs replicate within a host, the error-prone viral RNA replication machinery, generates an array of genetically related, yet distinct genomic variants, giving rise to intrahost diversity (Parameswaran et al., 2012, 2017).

## Morphological Diversity

It has become increasingly apparent that the DENV maturation process is far from complete as the virions exocytosed from the infected cell display a high degree of heterogeneity. This appears to be the result of incomplete prM cleavage, resulting in an entire spectrum of virion particles ranging from fully mature “smooth” virions (100% prM cleavage) to fully immature, “spiky” virions (0% prM cleavage). Partially mature virions contain varying proportions of “smooth” and “spiky” surfaces (Junjhon et al., 2008, 2010). Further diversity stems from the structural flexibility of the E proteins on DENVs, termed as “breathing,” which makes the virion structurally dynamic, with profound effects on epitope exposure. This is inferred from time- and temperature-dependent accessibility of certain virion epitopes to antibodies (Dowd et al., 2014; Kuhn et al., 2015).

## Antibodies Induced by DENVs

The immune responses to DENV infections are mainly targeted against structural proteins i.e., E and prM and one non-structural protein NS1 (Rey et al., 2018). The immune response against NS1 is prone to cross-react among all the DENV serotypes. Studies report that NS1 directly triggers vascular hyperpermeability by inducing pro-inflammatory vasoactive response via activation of Toll like receptor−4 (Modhiran et al., 2015) and disruption of endothelial glycocalyx (Puerta-Guardo et al., 2016; Slon-Campos et al., 2019). Apart from above, studies by (Chuang et al., 2014, 2016) reported the proposed participatory role of NS1 in severe DENV pathogenesis by promoting bleeding diathesis through inhibition of thrombin activity and enhancement of fibrinolysis. However, complete contrary results are also reported, where, mouse raised polyclonal NS1 antiserum or anti-NS1 mAbs protects mice from the lethal dose of DENV-2 and also reduces the vascular leakage (Beatty et al., 2015). Thus, further studies are required to delineate the role of NS1 associated outcomes during dengue virus infection. The immune response against the DENV structural proteins are summarized in Table 1. Natural DENV infections elicit both protective as well as pathogenic antibodies (Dejnirattisai et al., 2010; Chan et al., 2011). The pathogenic antibodies, which facilitate entry of non-neutralized and immature DENV into monocytes and macrophages, are essentially disease-spreading (DENV ADE promoting) antibodies. Investigations have revealed that prM and the FL epitope (FLE) are particularly immunodominant and elicit cross-reactive, disease-spreading antibodies (Beltramello et al., 2010; Slon Campos et al., 2018). An analysis of the memory B cell responses in DENV-infected individuals showed that ~60% of the human antibody response is directed toward the prM protein (Dejnirattisai et al., 2010). These are highly cross-reactive antibodies, which are capable of recognizing prM of all four DENV serotypes. Further, anti-prM-antibodies are poor neutralizers of DENV infectivity, but potent promoters of ADE (Beltramello et al., 2010; Dejnirattisai et al., 2010; Smith et al., 2012). These antibodies can actually make non-infectious immature virions infectious, by opsonizing them and facilitating their intracellular entry via the Fcγ receptor pathway. Once within the cell, these immature virions can undergo maturation and become inherently infectious, and spread to other cells. Likewise the anti-FLE antibodies (accounting for 20–30% of the antibody response to DENV), are also cross-reactive and tend to be weak neutralizers but strong promoters of ADE (Beltramello et al., 2010; Smith et al., 2012, 2014). The FLE, which is conserved among flaviviruses, is normally buried in the mature virion, but becomes accessible upon virus breathing (Cockburn et al., 2012; Pierson and Kuhn, 2012; Fibriansah et al., 2013). In the presence of prM and FLE antibodies, the partially immature and immature DENVs become fully infectious and increase the cellular viral load due to ADE (Halstead et al., 2010; Rodenhuis-Zybert et al., 2010) (Figure 1).

**Table 1:** Immune responses to DENV infections against the DENV structural proteins.

**Immunogenic DENV structural Antigens**	**Elicited human immune response**	**Antibody response (%)**	**Reference**
Membrane protein (prM)	Cross-reactive Non-neutralizing, disease enhancing antibodies, that enhance the ability of non-infective immature DENV to become infectious (ADE)	~60	Beltramello et al., 2010; Dejnirattisai et al., 2010; Smith et al., 2012
Envelope protein fusion loop epitope (FLE)	Cross-reactive neutralizing antibodies. These antibodies provide transient protection against the heterotypic DENVs, but seem to later (after 2–3 months) enhance replication of heterotypic DENVs (ADE)	20–30	Beltramello et al., 2010; Smith et al., 2012, 2014
DENV Envelope- dimer epitope (EDE)	EDE Ab cross-neutralizes all four DENV serotypes as well as ZIKV with low ADE potential	0–70^#^	Dejnirattisai et al., 2015; Barba-Spaeth et al., 2016; Slon-Campos et al., 2019
DENV Envelope host cell receptor binding domain (EDIII)	Type-specific highly potent DENV neutralizing antibodies, which seem to provide life-long homotypic protection with low ADE	5–10	Wahala et al., 2009; Chen et al., 2016

# *E-dimer epitope antibodies are highly variable in convalescent DENV plasma*.

Protective antibodies appear to be elicited by non-immunodominant epitopes, and are either type-specific or pan-DENV specific. The type-specific antibodies against DENVs are predominantly directed against EDIII (Crill and Roehrig, 2001), which is implicated in host cell receptor recognition and viral entry (Hung et al., 2004; Chin et al., 2007; Gromowski et al., 2008; Modis, 2014). Anti-EDIII antibodies constitute only 5–10% of the total immune responses in DENV infected individuals (Table 1) (Wahala et al., 2009). However, EDIII antibodies possess the highest DENV neutralizing capacity with minimal or no disease enhancing potential, when tested using *in vivo* dengue sensitive mouse models (Watanabe et al., 2015; Ramasamy et al., 2018).

**Figure 2:**
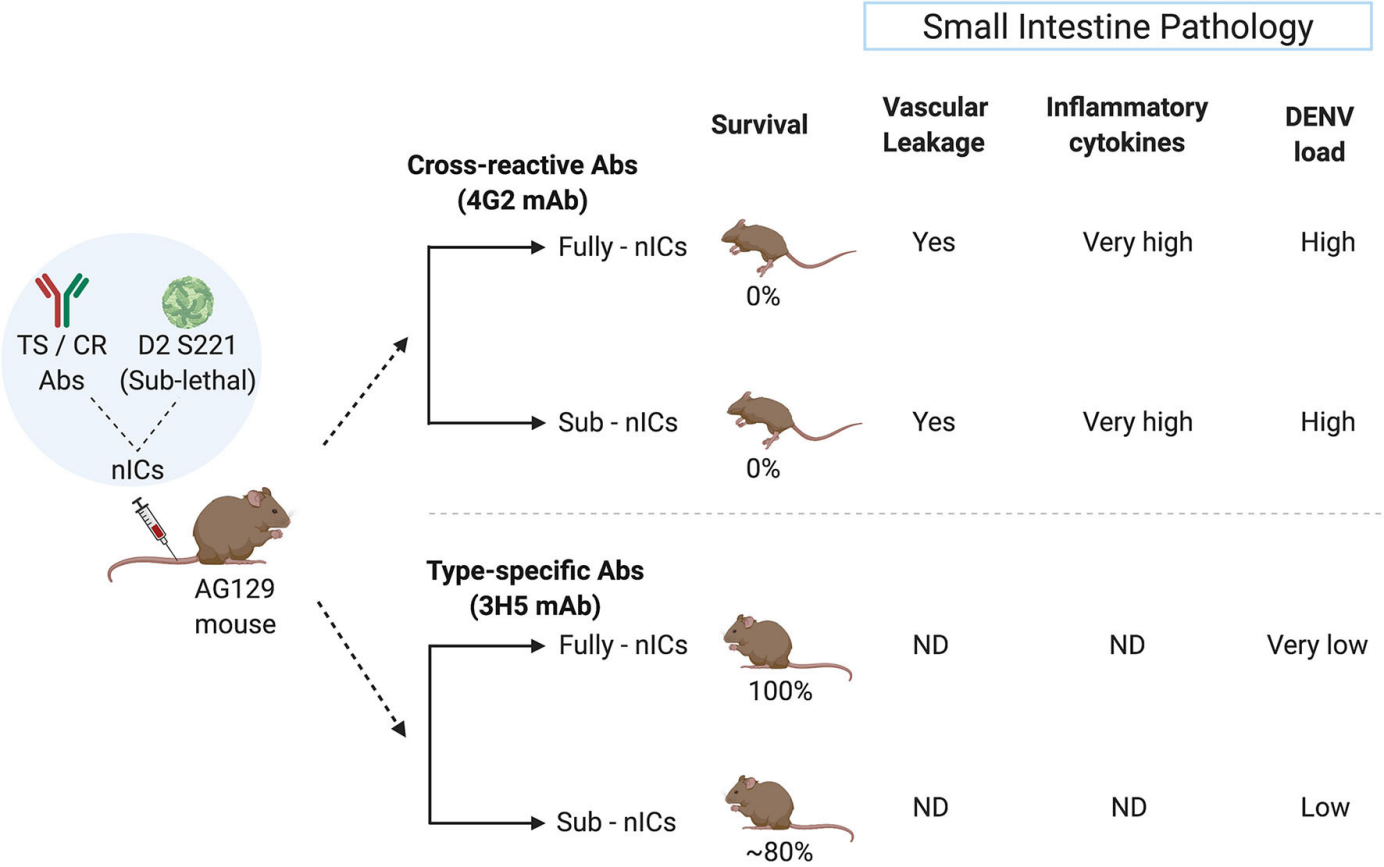
Type-specific (TS) monoclonal antibody (mAb) does not cause ADE in the mouse model whereas cross-reactive (CR) mAb does. The sublethal dose of D2 S221 was inoculated as fully- and sub-neutralized immune complexes (“Fully-nICs” and “Sub-nICs,” respectively) made with either cross-reactive (4G2) or type-specific (3H5) mAbs. Investigators (Watanabe et al., 2015) found 100% mortality and elevated levels of different ADE related parameters in the small intestine (Vascular leakage, inflammatory cytokines, and viral load) in both fully and sub-neutralized ICs made with 4G2. On the contrary, fully neutralized TS-ICs exhibited full protection accompanied by very low virus load. TS sub-neutralizing ICs showed a very minimal level of mortality accompanied by low virus load. ND, data not available (The illustrative figure created with Biorender.com).

Studies from the National University of Singapore demonstrate that the fully or partially neutralized immune complexes (ICs) of a mouse adapted DENV-2 strain (D2 S221) formed with DENV cross-reactive monoclonal antibody (mAb) against FLE (4G2) cause lethal disease in dengue sensitive AG129 mice (Watanabe et al., 2015). The high mortality was accompanied by intestinal pathology, vascular leak, increased cytokine storm, and small intestinal tissue virus load. However, fully or partially neutralized ICs of DENV-2 with type-specific EDIII mAb (3H5) showed protection and no disease enhancement under similar conditions (Watanabe et al., 2015) (Figure 2). This observation may explain the failure of Sanofi's vaccine to provide protection against DENV-2 in *in-vitro* assays despite exhibiting DENV-2 neutralizing activity.

Moreover, an anti-EDIII monoclonal antibody has been shown to be capable of protecting humanized mice from all symptoms associated with severe dengue (Robinson et al., 2015). Several potently protective mAbs isolated from DENV-infected individuals have been characterized. These mAbs recognize conformational epitopes, found only when E is displayed on the virion surface, and are known as quaternary epitopes. Many of these are serotype-specific (Teoh et al., 2012; Fibriansah et al., 2014, 2015a,b; Screaton et al., 2015). In addition, broadly neutralizing mAbs, which target conserved quaternary epitopes among the four DENV serotypes, have been identified recently (Dejnirattisai et al., 2015; Rouvinski et al., 2015). However, mimicking quaternary epitopes for a dengue vaccine candidate has been highly challenging. Recently, investigators have successfully displayed DENV neutralizing quaternary structures as covalently stabilized Envelope Dimer Epitopes (EDE) (Rouvinski et al., 2017; Thomas et al., 2020). Further studies show EDE antibodies to be dominant toward the infecting serotype with lower avidity against the other serotypes (Thomas et al., 2020) Interestingly, these EDE antibodies or EDE-Mab were able to cross-neutralize ZIKV infection *in vitro* and showed protection in a lethal mouse model (Swanstrom et al., 2016; Fernandez et al., 2017).

## DENV Cellular Immune Responses

Several excellent publications on cellular immune responses during DENV natural infection are available (Mathew and Rothman, 2008; Yauch et al., 2009; Zellweger et al., 2015; Elong Ngono et al., 2016; St. John and Rathore, 2019; Tian et al., 2019). Nonetheless, precise nature of a protective T cell response in the context of DENV infection and vaccination is yet to be delineated. Like the antibody response, T cell responses also are implicated both in protection as well as pathogenesis (Beaumier et al., 2008; Friberg et al., 2011; Weiskopf et al., 2013). However, unlike in the case of antibodies for which there is greater clarity on the nature of epitopes that elicit protective vs. pathogenic antibodies, similar information on T cell epitopes is not available. One of the key hypothesis to explain the sub-optimal performance of the Dengvaxia is that it had YFV specific T-cell epitopes and did not contain the DENV specific T-cell epitopes. Nonetheless, a few publications (Simmons et al., 2005; Weiskopf et al., 2013, 2015) indicate that YFV and DENV proteomes do carry several highly conserved cross-reactive T-cell epitopes in the NS3 and NS5 regions. Similar to the B-cell cross-reactive epitopes, the Dengvaxia and the other whole virus-based dengue vaccines do carry some level of cross-reactive T-cell epitopes. Thus, the sub-optimal performance of Dengvaxia cannot be solely attributed to the absence of DENV specific T-cell epitopes. Additional research is required to shed more light on the specific features of both arms of the adaptive immune system in the context of their opposing roles in protection and pathogenesis.

## Viral Interference

The success of the yellow fever LAV, based on the attenuated YFV variant YF17D (Monath, 2005; SAGE Working Group, 2013), provided the paradigm for a dengue LAV. Due to the ADE phenomenon, the dengue LAV needs to be tetravalent, so that it may provide immunity against all four DENV serotypes. However, mixing four monovalent dengue LAVs into a tetravalent formulation is associated with one component replicating better at the expense of the others, leading to a phenomenon known as viral interference (Dittmar et al., 1982; Edelman, 2011). Intuitively, one may posit a role for the dynamics of intra-host microevolution, referred to above, in viral interference. However, this remains to be experimentally ascertained.

Viral interference was historically noticed by Thai (Kanesa-Thasan et al., 2001; Sabchareon et al., 2002) and US Army (Edelman et al., 2003) researchers during initial attempts at developing tetravalent dengue LAVs. The consequence of such interference is that the immune response tends to be skewed toward one DENV serotype. This can lead to the situation wherein, though the LAV is a physically tetravalent mixture, it is essentially immunologically monovalent. The resulting partial protection can prime one to ADE in future, upon natural DENV exposure. In fact, the observation that Dengvaxia predisposed seronegative recipients to increased risk of severe dengue, starting at 3 years after the first dose (Hadinegoro et al., 2015), lends support to the occurrence of ADE (Halstead, 2017). The subsequent finding that Dengvaxia elicited antibodies predominantly specific to DENV-4 (Henein et al., 2017), is consistent with the vaccine having simulated a primary monotypic infection. It is to be expected that, like the natural DENVs, Dengvaxia, being a viral vaccine encoding prM and E, would also elicit predominantly prM- and FLE-specific antibodies, which are efficient ADE promoters. This would undoubtedly be true for other whole virus-based vaccines as well. This makes ADE evaluation a mandatory step during pre-clinical vaccine development.

Most live attenuated, killed or chimeric whole dengue virus-based vaccine candidates will mimic natural DENV infections and thus will elicit predominantly cross-reactive disease enhancing antibodies with limited type-specific protective antibodies. Therefore, there is a need for designing a dengue vaccine candidate that elicits predominantly protective antibodies (like anti-EDIII) in the absence of pathogenic antibodies (anti-prM and anti-FLE) (Lam et al., 2016; Screaton and Mongkolsapaya, 2018).

**Figure 3:**
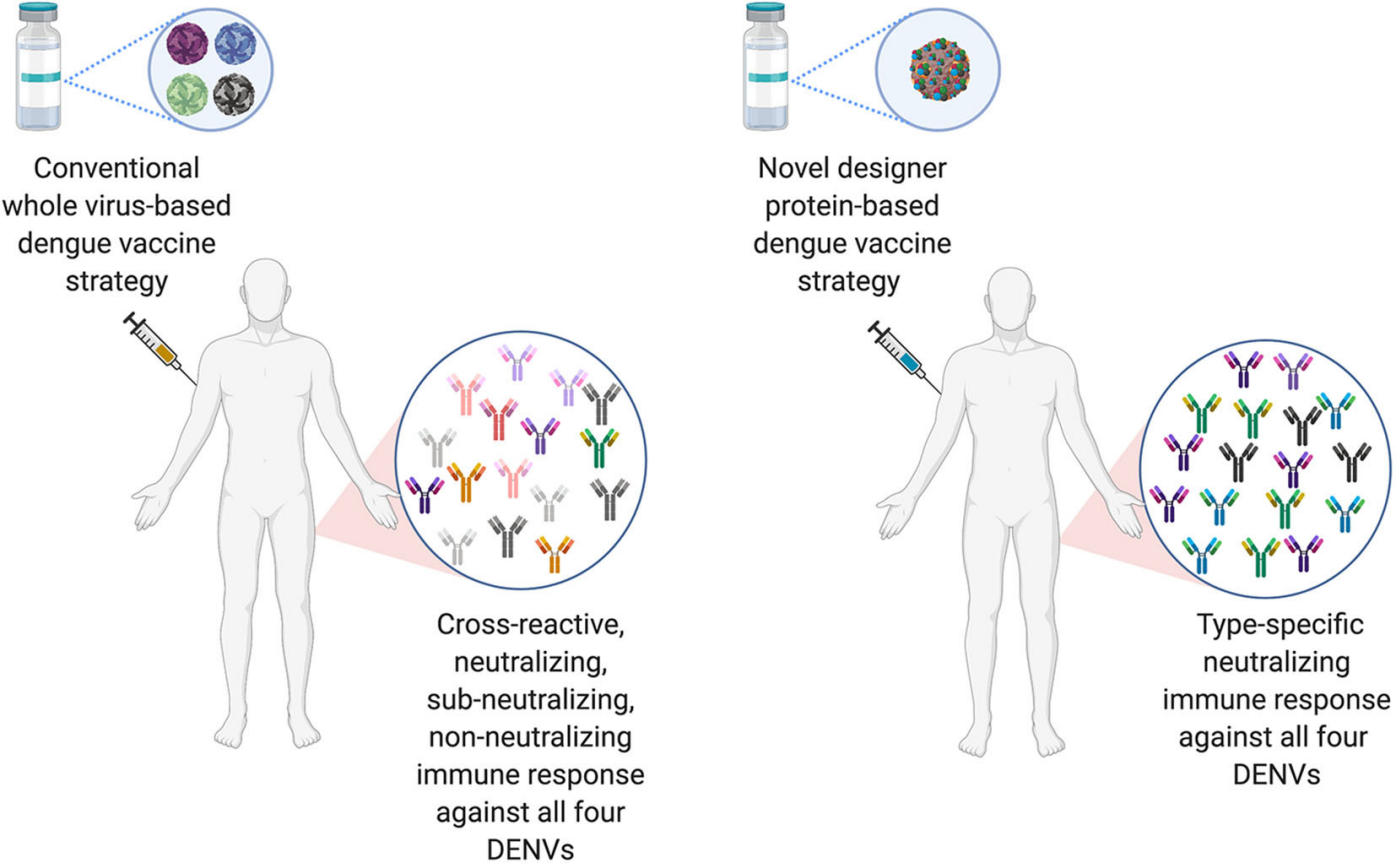
Comparison of conventional DENV vaccine strategy vs. Designer DENV vaccine candidate immune responses. Conventional DENV vaccine generally have the mixture of live-attenuated all four DENV serotypes, whereas, ongoing pre-clinical designer DENV vaccine candidate (s) has single entity expressing all four DENV component. The whole DENV-based vaccine inherently poses to induce imbalance immune responses with high load of cross-reactive Abs against all four DENV serotype, suggests to explore designer DENV vaccine strategy, which may induce non-enhancing type-specific neutralizing immune response in human (Figure designed with Biorender.com).

The ideal dengue vaccine should be tetravalent and generate long lasting, type-specific neutralizing antibodies against all the four-dengue virus serotypes. Therefore, EDIII seems like an ideal target for dengue vaccine development, as anti-EDIII antibodies have higher type-specific neutralization capacity with lower ADE potential (Ramasamy et al., 2018). Comparative immune responses of novel designer protein-based DENV vaccine strategy over the conventional whole virus-based DENV vaccines are highlighted in Figure 3.

A recent study on molecular simulations of dengue virus infection and experimental data suggest that the interplay between epitope accessibility, Ab specificity, Ab affinity, Ab concentration, and mature content of the virus significantly influence the degree of ADE (Ripoll et al., 2019). Since both Ab concentration and type specificity are critical host determinants of ADE, it is important to quantify not only the neutralizing antibody titer but also fine specificity (type-specific vs. cross-reactive) when assessing future dengue vaccine candidates. For the safety and efficacy of a vaccine, it is vital that the vaccine immunogen should be well-characterized However, the maturation states of dengue serotypes in a whole virus-based dengue may be difficult to control. Various maturation states of DENV serotypes may affect its ability to provoke protective Ab responses without creating conditions that increase the risk of severe dengue disease (Ripoll et al., 2019). Vaccine candidates capable of eliciting predominantly type-specific immune responses in the absence of FL and prM directed antibodies could exhibit higher virus neutralization with lower ADE potential (Ramasamy et al., 2018).

## ADE: *in vitro* vs. *in vivo* Assays

Often DENV infection-enhancing activity of antibodies is evaluated *in vitro* using cell lines expressing Fcγ receptors, such as THP-1, U937, or K562. These cell lines differ in their dengue-susceptibility, due to differences in the types and levels of Fcγ receptors expressed by them. To measure ADE, immune complexes (ICs), generated by pre-incubating DENV with the antibody, are added to these cells in culture, followed by measuring the amount of cellular DENV uptake. However, due to the diversity of these cell lines, the *in vitro* ADE assay is unlikely to reflect the true *in vivo* situation (Krol et al., 2019).

In this regard, ADE assays, based on dengue-sensitive mouse models, offer a closer approximation of the *in vivo* situation. The *in vivo* ADE assay is based on mice with genetic defects in the innate immune signaling pathway. Typically, an antibody whose ADE potential is to be evaluated, is either introduced into the mouse by passive transfer, followed by challenge with a sub-lethal dose of a mouse adapted DENV strain or pre-incubated with the mouse-adapted DENV strain to generate ICs, which are then injected into the mouse, followed by survival monitoring, as well as analysis of vascular leakage and cytokine production (Watanabe et al., 2015; Shukla et al., 2020).

The *in vivo* (mouse model) and *in vitro* (Fcγ receptor-bearing cell lines) ADE assays score differently when evaluated with cross-reactive vs. the type-specific DENV mAbs. This was evidenced in a recent study using the interferon α/β and γ receptor double knock-out AG129 mouse model (Watanabe et al., 2015). Highly neutralized ICs made by pre-incubating the pan-DENV cross-reactive FL-specific mAb 4G2 or EDIII DENV-2 type-specific mAb 3H5 and a mouse-adapted DENV-2 strain, did not manifest ADE in a THP-1 cell line-based *in vitro* ADE assay. However, unlike the ICs made with 3H5 mAb, the ICs generated with 4G2 mAb revealed potent lethality in the dengue-sensitive AG129 mouse, without enhancing serum viremia. This finding is further affirmed with a recent report published in EBioMedicine by Shukla et al. (2020). The investigators showed that both Dengue and Zika virus infections are enhanced by live attenuated dengue vaccine that majorly elicits FL and prM cross-reactive antibodies but not by a recombinant tetravalent Dengue Subunit Vaccine candidate, capable of inducing predominantly EDIII directed type-specific antibodies in the absence of antibodies to FL and prM epitopes in murine models. This study provides the first “head-to-head” experimental comparison of *in vivo* ADE potential between an approved yellow fever virus-based recombinant dengue vaccine “Dengvaxia” and a designed EDIII-based Dengue Subunit Vaccine Tetravalent (DSV4) candidate. In this study, investigators also included an “in-house” version of the whole DENV-based surrogate vaccine candidate by utilizing a physical mixture of tetravalent DENVs (TV DENV). The study revealed that antibodies elicited by Dengvaxia and TV DENV in BALB/c mice were predominantly cross-reactive and failed to offer protection against lethal DENV challenge in AG129 mouse model. Moreover, the completely neutralized immune complexes of DENV-2 made with the virus-based dengue vaccine sera promoted ADE of DENV-2 infection and caused mortality despite virus neutralization. On the other hand, DSV4-induced predominantly type-specific mouse antibodies not only provided significant protection against the lethal DENV-2 challenge but also did not promote ADE of DENV infection when evaluated in the AG129 mouse model. Anti-Dengvaxia and anti-TV DENV antibodies were directly associated in the elevation of intestinal pro-inflammatory cytokines (TNF-α & IL-6) production as well as viral load which leads to intestinal vascular leakage and ultimately death of mice. The study provides crucial insight that type-specific neutralizing Abs are vital for protection without ADE. Thus, testing *in vivo* ADE potential of neutralized DENV ICs in a small animal model offers a superior strategy to de-risk experimental dengue vaccine candidates during pre-clinical development.

## DENV/ZIKV Interaction and ADE

A key challenge in dengue vaccine development stems from the interaction between DENV and Zika virus (ZIKV), another human flaviviral pathogen transmitted by the same mosquito vector (Musso et al., 2015). More recently, concerns that DENV antibodies could enhance infection of ZIKV have been raised because: (1) ZIKV is phylogenetically related to DENV, (2) ZIKV outbreaks have occurred in DENV endemic regions around the world, and (3) DENV antibodies can enhance ZIKV infection both *in vitro* as well as *in vivo* in mice. Thus, the worry that a DENV vaccine could also enhance ZIKV disease in humans is a serious concern.

Studies show that the DENV-induced anti-FLE antibodies can interact with Fcγ receptors to mediate ZIKV uptake into susceptible cells. In fact, the recent ZIKV outbreaks associated with Guillain Barre syndrome in adults and microcephaly in infants, have occurred in regions of high DENV endemicity (Lessler et al., 2016; Culshaw et al., 2017), suggesting a role for ADE of ZIKV mediated by cross-reactive anti-DENV antibodies (Dejnirattisai et al., 2016; Bardina et al., 2017). This notion has received strong support from very recent work which shows that DENV-specific antibodies introduced into ZIKV-infected pregnant Stat2^−/−^ mice significantly increased placental damage, fetal growth restriction, and fetal resorption (Brown et al., 2019). Interesting findings were reported using the murine sera obtained by immunization with Dengvaxia, Tetravalent mixture of 1-4 DENVs (TV-DENV) and the recombinant DSV4 immunogens. The Dengvaxia and TV-DENV-based surrogate vaccine candidate anti-sera cross-neutralized ZIKV *in vitro* and also induced ADE of ZIKV infection in adult Stat2^−/−^ mice. Moreover, enhanced ZIKV infection were observed in several organs of Stat2^−/−^ mice inoculated with anti-Dengvaxia and anti-TV DENV sera. However, DSV4-induced anti-serum neither cross-neutralized ZIKV *in vitro* nor promoted ADE *in vivo* (Shukla et al., 2020). The pre-immunity against ZIKV is also a serious concern for developing the dengue vaccine. Recent Nicaraguan prospective pediatric cohorts study suggested that prior ZIKV infection can enhance severe dengue disease in future (Katzelnick et al., 2020).

## Designer Vaccines

It is becoming increasingly evident that alternate dengue vaccine strategies need to be explored (de Silva and Harris, 2018; Rey et al., 2018; Screaton and Mongkolsapaya, 2018), given the formidable challenges that are intrinsically associated with whole virus-based vaccines. It is quite obvious that successful dengue vaccines must be designed to target potently neutralizing epitopes (EDIII and quaternary epitopes such as EDE) while avoiding pathogenic epitopes (prM and FLE). Based on the recent knowledge on dengue virus biology and immunology, several dengue experts are veering to the view that a safe and effective dengue vaccine can be designed using recombinant DNA technology. These findings suggest that a designed EDIII-based VLP platform and stabilized E-Dimer Epitope (EDE)-based dengue vaccine could provide the basis for a safe and effective vaccine candidate capable of eliciting predominantly type-specific antibodies.

Recently, investigators have successfully engineered a stabilized EDE as a novel subunit DENV vaccine candidate. This has been achieved by interfacing the two E-monomers to form a E-dimer and lock this E-dimer via covalent disulphide linkages. The stabilized EDE protein was recognized by Mabs specific to the DENV quaternary epitopes. Moreover, the FLE was unavailable on the surface of these stabilized EDE, thus avoiding a significant level of cross-reactive antibodies to this FLE that are elicited by immunizations with E-monomers. This EDE design resulted in a higher level of serotype-specific immune responses as compared to the E-monomers (Thomas et al., 2020). The DENV cross-reactive antibodies elicited by EDE exhibited potent neutralization of all four DENV serotypes due to the conserved region of EDE (Barba-Spaeth et al., 2016; Fernandez et al., 2017; Rouvinski et al., 2017; Abbink et al., 2018; Thomas et al., 2020). Studies show that anti-dengue EDE mAbs exhibit protection against ZIKV infection in pregnant and non-pregnant immunocompromised C57BL/6 mice (Fernandez et al., 2017).

A recently published pre-clinical results of a virus-like particle (VLP) vaccine candidate known as DSV4 (**D**engue **S**ubunit **V**accine **Tetra**valent) are very promising (Ramasamy et al., 2018). This candidate is based on EDIII. Unlike domains EDI and EDII, which elicit largely flavivirus cross-reactive and weakly-neutralizing or non-neutralizing antibodies, EDIII elicits potent serotype-specific virus-neutralizing antibodies. The “four-in-one,” tetravalent vaccine candidate incorporates the EDIIIs of all four DENVs spliced together through flexible linkers in a single translational reading frame. Further, it is genetically fused with Hepatitis-B surface antigen (HBsAg) and co-expressed with four expression cassettes of HBsAg in order to display EDIIIs on the surface of HBsAg virus-like-particles (VLPs).

**Figure 4:**
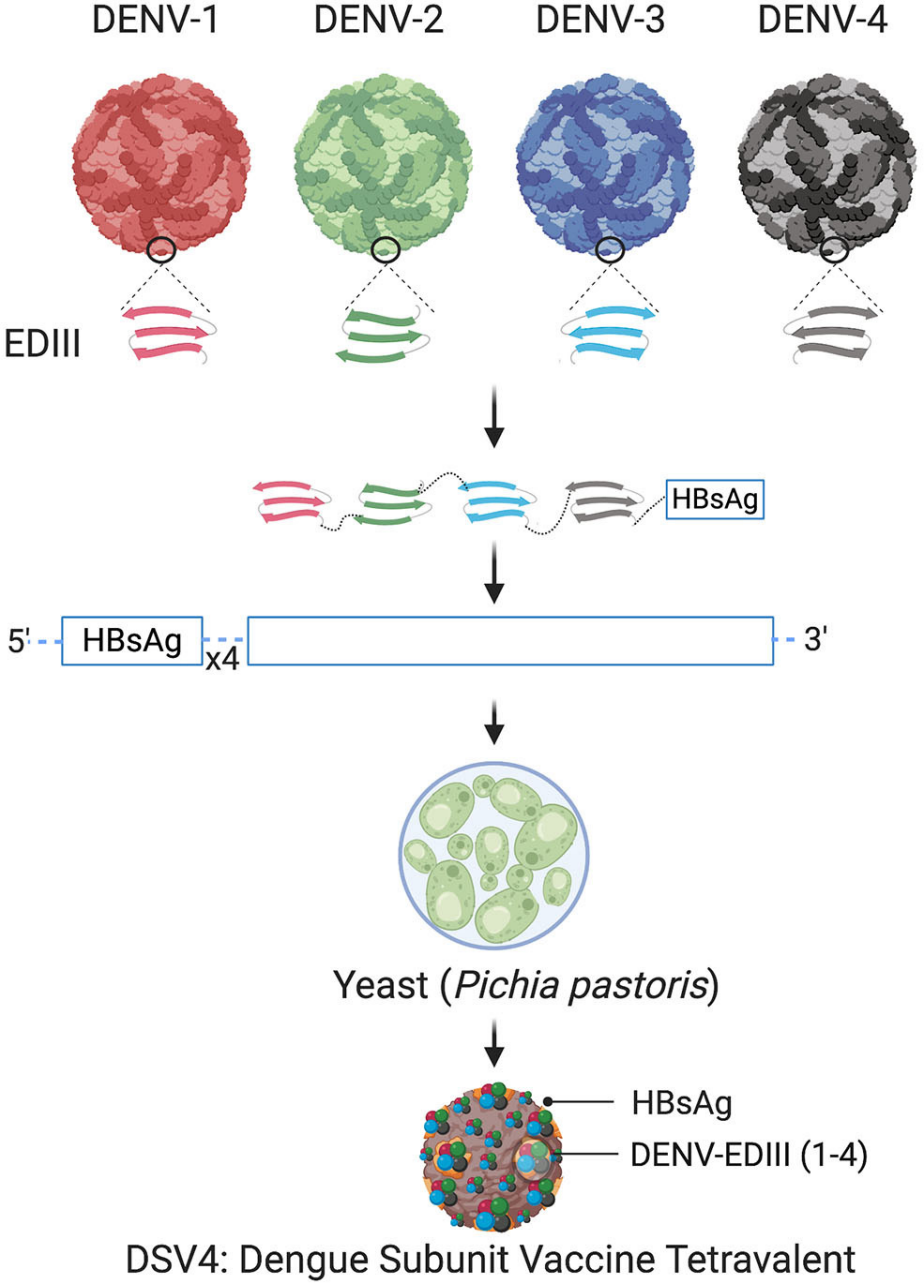
Design of DSV4. Top panel shows schematic diagram of all four DENV, encircled parts represent envelope domain III (EDIII) component of all four viruses. These EDIIIs components were linked with hexa-glycine linkers, fused to n-terminus of Hepatitis-B surface antigen (HBsAg) and further cloned in the background of four copies of HBsAg. The cloned expression cassette including DENV-EDIIIs, was integrated into the yeast expression host, *Pichia pastoris*, and DSV4 antigen was purified from the recombinant host for further immunological studies. The figure approach is adapted from Ramasamy et al. (2018) and created with Biorender.com.

The schematic representation of DSV4 design is shown in Figure 4. DSV4 assembles into VLPs and displays critical DENV neutralizing epitopes of all 4 serotypes. It is immunogenic in mice and macaques with aluminum hydroxide as adjuvant. It elicits serotype-specific neutralizing antibodies against all four DENVs in mice. These antibodies exhibit breadth of neutralization against various genotypes of each serotype (Ramasamy et al., 2018). The lack of *in vivo* ADE in the recombinant DSV4 design is a crucial differentiator from the whole virus-based dengue vaccine strategies. Table 2 compares the key features of DSV4 with other whole DENV-based vaccine strategies.

**Table 2:** Comparison of designer dengue vaccine candidate over whole DENV-based vaccine or vaccine candidates.

**Features**	**Whole DENV-based vaccine/candidate (s)**	**Designer dengue Vaccine Candidate**	
		**DSV4**	**E-dimer**
Immunogen	Mix of 4 viruses	4-in-1 VLP	Quaternary epitope
Viral interference	Yes	Not applicable	Not applicable
Viral breathing	Yes	Not applicable	Not applicable
FLE,	Present	Absent	Buried
prM, NS1	Present	Absent	Absent
Type-specific Abs responses	Minimal	Maximum	Fair
*In vivo* DENV ADE	High	Absent	Low
*In vivo* ZIKV ADE	Present	Absent	Low
Expression host	Mammalian cells	Yeast	Transient mammalian cells

## Conclusions

Antibodies elicited by DENVs play roles both in protection against, and pathogenesis of, dengue disease. Protective immunity is determined by the balance between these two opposing antibody roles. A safe and efficacious dengue vaccine must confer durable protection against all four DENV serotypes, without the risk of ADE. Further, it should be suitable for all age groups, irrespective of pre-vaccination serostatus. LAVs have been the focus of most efforts, with one partly protective vaccine already licensed, one which has completed Phase 3 trials and a third one due to complete efficacy trials soon. However, the unique features of DENVs and the predominantly ADE-prone nature of antibodies they elicit, coupled to the issues of viral interference rendering physically tetravalent LAVs, functionally monovalent, could continue to pose a challenge for a risk-free dengue vaccine. In this regard, recombinant subunit vaccine strategies that can facilitate selective retention of neutralizing epitopes, while eliminating ADE-associated epitopes, offer alternate promising options that must be explored. Subunit vaccines for preventing infections by other viruses such as hepatitis B, hepatitis E and human papilloma viruses have been licensed (Shukla et al., 2019). Learning from the Dengvaxia experience, it is very critical to assess the *in vivo* ADE potential of all vaccine candidates early on, using the available dengue-sensitive mouse ADE model. The safety and efficacy are inter-linked attributes and both must be evaluated.

## Author Contributions

All authors worked on the study conception and design, analyzed, and interpreted the data, read, and approved the submitted version.

## Conflict of Interest

The authors declare that the research was conducted in the absence of any commercial or financial relationships that could be construed as a potential conflict of interest.
